# Relationship between SpO_2_/FiO_2_ and PaO_2_/FiO_2_ in patients with acute respiratory distress syndrome: a single-center, retrospective cohort study from Xining, China

**DOI:** 10.3389/fmed.2026.1774390

**Published:** 2026-05-07

**Authors:** Jian Sun, Jing Zhang, Litao Guo, Xiaoqin Liu

**Affiliations:** 1 School of Clinical Medicine, Qinghai University, Xining, Qinghai, China; 2 The First Affiliated Hospital of Xi’an Jiaotong University, Xi'an, China; 3 Department of Critical Care Medicine, Qinghai Provincial People’s Hospital Xining, Xining, Qinghai, China

**Keywords:** acute respiratory distress syndrome, oxygen saturation, PaO_2_/FIO_2_ ratio, partial oxygen pressure, SpO_2_/FiO_2_ ratio

## Abstract

**Background:**

The updated global criteria for diagnosing acute respiratory distress syndrome (ARDS) include SpO_2_/FiO_2_ as a substitute for PaO_2_/FiO_2_ to enhance practicality in settings with limited resources and reduce reliance on arterial blood gas testing. However, the applicability of these SpO_2_/FiO_2_ thresholds in high-altitude regions remains unvalidated. This study investigated the relationship between the SpO_2_/FiO_2_ and PaO_2_/FiO_2_ in patients with ARDS in the Xining region of Qinghai and validated whether the critical values for hypoxemia severity classification based on SpO_2_/FiO_2_ are applicable in this population. A retrospective analysis was conducted on the clinical data of 276 patients with ARDS admitted to the intensive care unit (ICU) of Qinghai Provincial People’s Hospital from October 2020 to October 2024. All patients who met the inclusion criteria were classified into mild, moderate, and severe ARDS as defined by the Berlin Definition. Spearman’s correlation analysis was used to assess the correlation between SpO_2_/FiO_2_ (corrected) and PaO_2_/FiO_2_ (corrected). Curve estimation was used to derive the linear and nonlinear relationships between SpO_2_/FiO_2_ and PaO_2_/FiO_2_, as well as between SpO_2_/FiO_2_ (corrected) and PaO_2_/FiO_2_ (corrected). Receiver operating characteristic curve analysis was used to validate whether the cutoff values proposed by the new global definition for SpO_2_/FiO_2_-based hypoxemia severity stratification apply to Xining.

**Results:**

A significant nonlinear relationship was observed between the SpO_2_/FiO_2_ and PaO_2_/FiO_2_ ratios. The optimal fitting model was a power function model: SpO_2_/FiO_2_ (corrected) = 5.661 × [PaO_2_/FiO_2_ (corrected)]^0^·^71^. The area under the curve (AUC) of the SpO_2_/FiO_2_(corrected) for predicting mild ARDS was 0.912, with an optimal diagnostic threshold cutoff value of 360.4. For moderate ARDS, the AUC was 0.487, with a cutoff value of 238.8. For severe ARDS, the AUC was 0.956, with an optimal cutoff value of 168.

**Conclusion:**

This study revealed a nonlinear relationship between the SpO_2_/FiO_2_ and PaO_2_/FiO_2_ ratios in patients with ARDS in Xining, Qinghai Province, a characteristic that may better align with the pathophysiological features of high-altitude regions. The globally redefined hypoxemia grouping thresholds based on SpO_2_/FiO_2_ may not apply to this area, suggesting that regional diagnostic criteria should be established according to altitude variations.

## 1 Background

Acute respiratory distress syndrome (ARDS) is an acute, diffuse, inflammatory lung injury triggered by pneumonia, infection, trauma, blood transfusion, burns, aspiration, or shock (1). The 2012 Berlin Definition advanced clinical standardization by harmonizing diagnostic criteria; however, it explicitly excluded patients receiving noninvasive ventilation (NIV) or high-flow nasal cannula (HFNC) therapy—respiratory support modalities whose clinical application increased substantially during and after the COVID-19 pandemic (2). Before updating the new global definition guidelines for ARDS, the diagnosis of ARDS required the use of arterial blood to calculate the ratio of arterial oxygen partial pressure to oxygen concentration (PaO_2_/FiO_2_). However, this method is invasive and expensive, making it infeasible in many resource-limited settings (3). Therefore, clinicians have long sought a rapid and simple method for assessing oxygenation in patients with ARDS (4).

To address this limitation, the global revised definition introduces key updates to the ARDS criteria, formally incorporating the pulse oximetry saturation-to-oxygen concentration ratio (S/F; SpO_2_/FiO_2_ with SpO_2_ ≤ 97%) as an alternative indicator to the PaO_2_/FiO_2_ (P/F) ratio for precise identification of hypoxemia. The diagnostic framework also incorporates the use of NIV, HFNC, and lung ultrasound, moving beyond the traditional reliance on arterial blood gas analysis and imaging studies (2, 5). This revision enhances the ability to identify patients with ARDS in resource-constrained settings and provides crucial clinical guidance by expanding the indications for early intervention, thereby facilitating the timely initiation of treatment (2, 5).

Healthy individuals residing at high altitudes experience lower oxygen partial pressures than those living at sea level. Long-term inhabitants of plateau regions develop a greater tolerance to hypoxia; however, individual variations also contribute to differing levels of hypoxic adaptation. Consequently, the pathophysiological changes observed in patients with ARDS in plateau areas differ from those noted at lower altitudes (3, 6). The Berlin Definition states that at medium to high altitudes above sea level, the biological significance of a given PaO_2_/FiO_2_ ratio may differ from that at sea level, and proposes a mathematical formula for adjusting the PaO_2_/FiO_2_ ratio according to altitude (7–10). If the altitude is > 1,000 m, the following correction formula is applied: (PaO_2_ or SpO_2_)/FiO_2_ × (actual atmospheric pressure/760) (1). In a 2007 comparison of SpO_2_/FiO_2_ and PaO_2_/FiO_2_ ratios among patients with ARDS, derived from a dataset of 2,613 measurements, the relationship between S/F and P/F was: S/F = 64 + 0.84 × (P/F) (*p* < 0.0001; *r* = 0.89) (11). The SpO_2_/FiO_2_ and PaO_2_/FiO_2_ ratios exhibit a strong correlation, particularly when the PaO_2_/FiO_2_ ratio is < 300. The new definition similarly employs this linear equation to establish the critical threshold for SpO_2_/FiO_2_ (1, 12, 13). Several studies have established the correlation and efficacy of SpO_2_/FiO_2_ and PaO_2_/FiO_2_ ratios in mechanically ventilated patients at an altitude of 2,600 meters (14). However, the validity of the SpO_2_/FiO_2_ threshold for the diagnosis of ARDS requires further investigation (15). Furthermore, no studies have investigated the relationship between changes in PaO_2_ and SpO_2_ in patients with ARDS. Currently, no domestic research exists in this area, and although international studies have been conducted, none have been performed in high-altitude regions (16).

This study aimed to investigate the relationship between SpO_2_/FiO_2_ and PaO_2_/FiO_2_ in patients with ARDS in Xining, Qinghai Province (elevation 2,261 m^1^), to determine whether the diagnostic thresholds for SpO_2_/FiO_2_ severity in the new definition are applicablepply to this region. The objective of the study was to enable the rapid, non-invasive screening of patients with ARDS in Xining and to provide a simple and feasible method for assessing disease severity in this population (16).

## 2 Methods

### 2.1 Study design and ethical approval

This single-center retrospective study sought to examine the relationship between SpO_2_/FiO_2_ and PaO_2_/FiO_2_ in patients with ARDS in Xining, Qinghai Province, and to assess whether the SpO_2_/FiO_2_-based severity thresholds proposed in the new global definition are applicable in this region. This study utilised retrospective data and was granted exemption from obtaining informed consent by the Research Ethics Review Committee of Qinghai Provincial People’s Hospital (approval number: 2025–086-03). All patients who met the inclusion criteria were assessed for hypoxemia using the Berlin Definition (PaO_2_/FiO_2_ ratio). Patients were stratified into mild, moderate, and severe ARDS groups according to disease severity. Further classification was performed based on the ARDS Global Definition, classifying the patients into intubated and non-intubated groups according to their mode of ventilator support.

### 2.2 Inclusion and exclusion criteria

The inclusion criteria were as follows: (a) met the diagnostic criteria for ARDS as proposed by the 2012 Berlin Definition; (b) aged ≥ 18 years; and (c) individuals who have lived in high-altitude areas for generations (born and raised in Xining, or with continuous residency >10 years, as documented in medical records). The exclusion criteria were as follows: (a) respiratory failure explained by cardiac dysfunction or fluid overload (as assessed by echocardiography) (1); (b) patients with an intensive care unit (ICU) stay of < 24 h (9) (c) individuals residing at altitudes exceeding 3,000 m, whether migrants or indigenous populations, exhibiting red blood cell counts exceeding 6.5 × 10^12^/L, with hemoglobin levels exceeding 210 g/L for males and 190 g/L for females (17); (d) individuals who have lived long-term at low altitudes with acute respiratory failure occurring within 24 h to 7 days after rapid ascent to high altitude (17); and (e) patients with missing data.

### 2.3 Study population and environment

**Figure 1:**
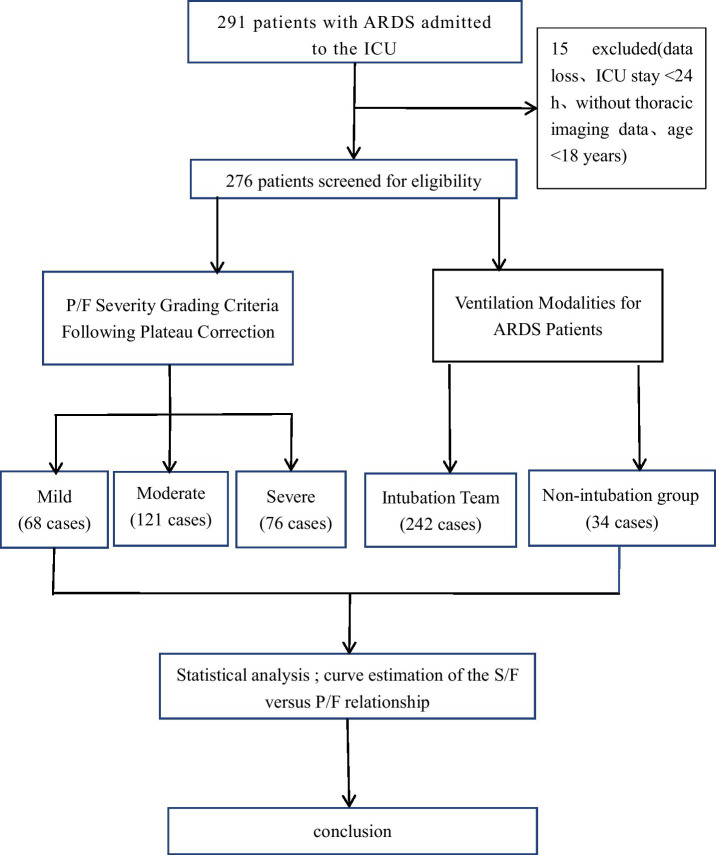
Patient Screening and Grouping Flowchart; P/F, PaO_2_/FiO_2_ ratio; ARDS, acute respiratory distress syndrome.

Clinical data were collected from 276 patients with ARDS admitted to the ICU of Qinghai Provincial People’s Hospital between October 2020 and October 2024. Data included the first blood gas analysis upon ICU admission, general information (age, sex, height, weight, and underlying conditions), clinical and laboratory examination indicators (ventilator parameters, hemoglobin, hematocrit, bilirubin, creatinine levels, white blood cell count, platelet count, C-reactive protein, lactate levels, and other relevant indicators), APACHE II score, SOFA score, SIRS score, and CURB-65 score. Patients’ initial blood gas data were obtained using the ABL90 FLEX blood gas analyzer (Radiometer Medical Equipment, Shanghai, Limited Liability Company, serial numbers R1075N043 and R1092N014). The Henderson–Hasselbach equation ([H+] = 24 × PaCO_2_/HCO₃^−^) was used to assess blood gas consistency, and all included blood gas samples passed the consistency test. All SpO_2_ data were obtained from continuous pulse oximetry monitoring, with readings recorded concurrently with the first arterial blood gas analysis (within a 5-min window) following ICU admission. Monitoring was performed using a patient monitor (Philips [China] Investment Limited Liability Company; product number YZB/GER5891-2014) equipped with the manufacturer’s standard SpO_2_ sensor (M1191B/M1942A). The sensor was preferentially placed on the middle finger of the non-dominant hand. A stable SpO_2_ value was recorded only after confirming a reliable signal, defined by the consistency between the oximeter heart rate and the ECG heart rate, alongside a regular plethysmographic waveform, for a period exceeding 30 s. The recorded SpO_2_ value represents a single stable reading taken immediately prior to the blood draw, rather than an average of multiple measurements. According to the Berlin Definition for ARDS severity classification, 265 patients met the PaO_2_/FiO_2_ ratio plateau correction group criteria, with 68 in the mild group, 121 in the moderate group, and 76 in the severe group. Grouping according to ventilation mode yielded 242 intubated and 34 non-intubated patients (Figure 1).

### 2.4 Statistical analyses

Data processing was performed using SPSS 26.0 statistical software. The one-sample Kolmogorov–Smirnov test (two-tailed) was used to assess whether the quantitative data met the assumption of normal distribution. For normally distributed quantitative data, results are presented as the mean ± standard deviation (*x̄* ± *s*). Comparisons between two groups were analyzed using the t-test, whereas comparisons among three or more groups were analyzed using repeated measures analysis of variance (ANOVA). Non-normally distributed quantitative data are presented as the median (*Q*_25_, *Q*_75_). Comparisons between two groups were performed using the Mann–Whitney U test, whereas comparisons among three or more groups were analyzed using Kruskal–Wallis one-way ANOVA (K samples). Categorical data were analyzed using four-field tables or row × column χ^2^ tests. Spearman’s correlation analysis was used to examine the relationship between SpO_2_/FiO_2_ and PaO_2_/FiO_2_. Subsequently, curve estimation was conducted to establish a relationship model and parameter estimation for SpO_2_/FiO_2_ (dependent variable) and PaO_2_/FiO_2_ (independent variable). Curve estimation was also performed on SpO_2_/FiO_2_ (corrected; dependent variable) and PaO_2_/FiO_2_ (corrected; independent variable) to establish a relationship model and derive parameter estimates, thereby obtaining the correlation equation. *p* < 0.05 was considered significant.

## 3 Results

### 3.1 Data comparison of different severity grades under the newly defined P/F plateau correction standard group

A total of 265 patients met the PaO_2_/FiO_2_ plateau correction criteria, of which 68 (25.7%), 121 (45.7%), and 76 (28.6%) were classified into the mild, moderate, and severe ARDS groups, respectively. The most common risk factor was pneumonia (*n* = 151, 56.9%), followed by extrapulmonary infection (*n* = 64, 24.2%). The proportion of patients with mild (18.5%) and severe ARDS (33.1%) among the pneumonia-related cases was slightly lower than that among the infection-related cases. However, the combined proportion of moderate and severe ARDS attributable to pneumonia-related factors was 81.5% (48.3% + 33.1%). Significant differences were observed between the severity grades of ARDS caused by different risk factors (pneumonia, infection, aspiration, and severe trauma) (*p* = 0.001). Patients with severe ARDS had higher ventilator settings than those with mild or moderate ARDS. The more severe the ARDS, the higher the ventilator settings for FiO_2_ and positive end-expiratory pressure (PEEP) levels, with significant differences (*p* < 0.05). Significant differences were observed in PaO_2_, PaO_2_/FiO_2_, and corrected PaO_2_/FiO_2_ among the three patient groups (*p* < 0.05). Patients with more severe ARDS exhibited lower PaO_2_ levels in arterial blood gas analysis, as well as lower oxygenation indices and corrected oxygenation indices. However, no significant differences were observed in pH, PaCO_2_, and lactate levels. Compared with patients with mild or moderate ARDS, those with severe ARDS exhibited significant differences in SpO_2_/FiO_2_ measured by pulse oximetry, SpO_2_/FiO_2_ (corrected), C-reactive protein, and creatinine levels (*p <* 0.05). Patients with severe ARDS exhibited higher C-reactive protein levels and significantly lower SpO_2_/FiO_2_ and SpO_2_/FiO_2_ (corrected) levels. Red blood cell count, white blood cell count, platelet count, hemoglobin, hematocrit, total bilirubin, cystatin C, and blood urea nitrogen levels showed similar values across the three groups, with no significant differences (*p*>0.05). Patients with severe ARDS had slightly higher SOFA and SIRS scores in disease assessment than those with mild and moderate ARDS, with significant differences among the three groups (*p* < 0.05). The APACHE II scores, CURB-65 scores, and ICU length of stay were similar across the three groups, with no significant differences (*p*>0.05). Among the three patient groups, 115, 104, and 100 patients had hypertension, chronic pulmonary insufficiency, and chronic cardiac insufficiency, respectively. The moderate ARDS group exhibited a higher prevalence of diabetes mellitus (*n* = 28, 45.1%), hypertension (*n* = 53, 46.1%), chronic pulmonary insufficiency (*n* = 48, 46.2%), and chronic heart failure (*n* = 50, 50%) (Table 1).

**Table 1:** General characteristics of different severity groups under P/F plateau correction criteria, *n* (%).

Variables	P/F Mild (*n* = 68)	P/F Moderate (*n* = 121)	P/F Severe (*n* = 76)	H/*χ^2^* value	*p* value
Demographic characteristics					
Age (years)	67 (53.5, 75)	69 (57.5, 78)	69 (60, 75.75)	0.967	0.617
Height (cm)	167.5 (160, 173.75)	166 (160, 171)	168 (160, 171.75)	0.356	0.837
Weight (kg)	62 (55.25, 70)	65 (60, 71.75)	60 (51, 70)	4.881	0.087
Gender					
Male (*n* = 182)	42 (23.1)	86 (47.3)	54 (29.7)	2.033^a^	0.362
Woman (*n* = 83)	26 (31.3)	35 (42.2)	22 (26.5)		
Respiratory parameters					
FiO_2_ (%)	40 (40, 45)	60 (50, 70)	100 (80, 100)	171.714	<**0.001**
PEEP (cmH_2_O)	5 (1, 5)	5 (5, 6)	6 (5, 8)	44.337	<**0.001**
Blood gas analysis					
pH	7.40 (7.35, 7.44)	7.40 (7.32, 7.46)	7.37 (7.27, 7.44)	3.945	0.139
PaO_2_ (mmHg)	76 (69.67, 84.22)	64.6 (56, 74.85)	53.75 (45.82, 60.37)	99.451	<**0.001**
PaCO_2_ (mmHg)	36.85 (32.02, 40.97)	36.5 (31, 48)	37.9 (30, 48)	1.24	0.538
Lactic acid (mmol/L)	1.9 (1.22, 3.0)	1.8 (1.2, 2.9)	2.1 (1.42, 3.9)	5.246	0.073
Oxygenation Index (first time)	179.25 (167.52, 198.87)	114.2 (93.4, 132.5)	61.05 (51.57, 69.6)	224.018	<**0.001**
Oxygenation index (corrected)	233 (217.8, 257.5)	148.4 (121.45, 172.2)	78.9 (66.33, 90.45)	228.186	<**0.001**
Laboratory indicators					
SpO_2_/FiO_2_ (Pulse Oximeter)	228.25 (201.1, 237.37)	155 (129.25, 182)	90 (82, 107.12)	179.467	<**0.001**
SpO_2_/FiO_2_ (corrected)	296.7 (261.42, 308.52)	201.5 (168, 236.6)	117 (106.6, 139.2)	179.514	<**0.001**
Red blood cell count (×10^12^/L)	4.06 (3.28, 4.76)	4.22 (3.23, 5.28)	4.30 (3.26, 4.81)	1.902	0.386
White Blood Cell Count (×10^12^/L)	10.34 (7.26, 15.28)	9.98 (7.07, 15.22)	10.29 (6.98, 15.18)	0.075	0.963
Platelet count (×10^12^/L)	127.5 (81.75, 173.75)	152 (96, 221.5)	131.5 (88.5, 186)	4.95	0.084
Hemoglobin (g/L)	122.5 (97.25, 147.25)	130 (101, 159)	128.5 (102, 146)	0.924	0.63
Hematocrit (%)	37.55 (29.85, 43.82)	38.7 (29.1, 49.8)	38.15 (30.45, 45.45)	1.409	0.494
Total Bilirubin (μmol/L)	18.45 (12.37, 28.15)	21.4 (15.03, 32.5)	19.65 (12.97, 32.3)	2.643	0.267
C-reactive protein (mg/L)	7.70 (1.39, 16.54)	10.58 (4.73, 22.67)	16.20 (7.10, 25.94)	18.068	<**0.001**
Renal function indicators					
Creatinine level (μmol/L)	99.5 (75.25, 135.5)	81 (62.5, 116)	81 (67.25, 132.75)	7.348	**0.025**
Cystine (mg/L)	1.25 (0.94, 2.30)	1.2 (0.99, 1.8)	1.38 (1.02, 1.93)	1.625	0.444
Blood Urea Nitrogen (mmol/L)	8.89 (6.36, 14.15)	8.09 (5.33, 14.70)	9.04 (6.60, 12.64)	0.977	0.614
Disease scores					
APACHEII scores	22.5 (17, 26.75)	22 (17, 28.5)	25 (18, 31)	3.022	0.221
SOFA scores	8 (6.25, 10)	8 (5, 10)	9 (6, 12)	7.905	**0.019**
SIRS scores	2 (1, 2)	2 (1, 3)	2 (2, 3)	10.287	**0.006**
CURB-65 scores	2 (2, 3)	2 (2, 3)	2 (2, 3)	4.826	0.09
Hospitalization status					
ICU length of stay	7 (3, 15.75)	7 (3, 17)	5.5 (3, 15)	0.918	0.632
Risk factors for etiology					
Pneumonia (*n* = 151)	28 (18.5)	73 (48.3)	50 (33.1)	22.653^a^	**0.001**
infection (*n* = 64)	17 (26.6)	25 (39.1)	22 (34.4)		
Aspiration (*n* = 6)	4 (66.7)	2 (33.3)	0 (0)		
Severe trauma (*n* = 44)	19 (43.2)	21 (47.7)	4 (9.1)		
Underlying medical conditions					
Diabetes (*n* = 62)	16 (25.8)	28 (45.1)	18 (29.1)	26.22^a^	0.926
Hypertension (*n* = 115)	32 (27.8)	53 (46.1)	30 (26.1)		
Chronic pulmonary insufficiency (*n* = 104)	27 (25.9)	48 (46.2)	29 (27.9)		
Chronic heart failure (*n* = 100)	22 (22)	50 (50)	28 (28)		
None (*n* = 51)	14 (27.5)	20 (39.2)	17 (33.3)		

The elevation of the Xining area is 2,261 m; Atmospheric pressure is approximately 581 ~ 582 mmHg; Perform calibration for PaO_2_/FiO_2_ or SpO_2_/FiO_2_ × 1.3; pH is the acidity or alkalinity level; PaO_2_ is the partial pressure of oxygen in arterial blood; PaCO_2_ is the partial pressure of carbon dioxide in arterial blood; SpO_2_ denotes blood oxygen saturation; FiO_2_ denotes inspired oxygen concentration; PEEP denotes positive end-expiratory pressure; the oxygenation index denotes PaO_2_/FiO_2_; Oxygenation index (corrected): PaO_2_/FiO_2_ (corrected); APACHE II scores (Acute Physiology and Chronic Health Evaluation II); SOFA scores (Sequential Organ Failure Assessment); SIRS scores (Systemic Inflammatory Response Syndrome, SIRS); CURB-65 Scores (CURB-65 Pneumonia Severity Scores);a for row × list χ^2^ test.

The bold values indicate *p* < 0.05.

### 3.2 Comparison of general patient characteristics across different ventilation mode groups

Among the different ventilation mode groups, the intubated group consisted of 242 patients, whereas the non-intubated group comprised 34 patients. Intubated patients with ARDS had significantly higher ventilator settings for FiO_2_ and PEEP levels (*p* < 0.05) than non-intubated patients with ARDS. Patients in the intubated group exhibited significant differences in pH and PaCO_2_ compared to the non-intubated group (*p* < 0.05), with lower pH levels and higher PaCO_2_ levels. Indicators reflecting patient oxygenation status, such as PaO_2_/FiO_2_, PaO_2_/FiO_2_ (corrected), SpO_2_/FiO_2_, and SpO_2_/FiO_2_ (corrected), showed significant differences between the intubated and non-intubated groups (*p* < 0.05). Patients in the intubated group exhibited lower oxygenation indicators than non-intubated patients with ARDS. Significant differences were observed between the intubated ARDS group and the non-intubated group regarding red blood cells, white blood cells, hemoglobin, hematocrit, and total bilirubin (*p* < 0.05). Intubated patients with ARDS had slightly higher SOFA and SIRS scores on disease severity assessments than non-intubated patients with ARDS, with significant differences between the two groups. Both groups exhibited similar APACHE II scores, CURB-65 scores, and ICU length of stay, with no significant differences (Table 2).

**Table 2:** General information on groups with different ventilation modes, *n* (%).

Variables	Intubation team (*n* = 242)	Non-intubation group (*n* = 34)	*Z*/*χ^2^*value	*p* value
Demographic characteristics				
Age (years)	67 (58, 75)	73 (56, 81)	3411.500	0.107
Height (cm)	167 (160, 172)	165 (157, 172)	3712.000	0.355
Weight (kg)	65 (55, 70)	64 (57, 70)	4049.500	0.882
Gender				
Male (*n* = 186)	166 (89.2)	58 (10.8)	1.295^a^	0.255
Woman (*n* = 90)	76 (84.4)	14 (15.6)		
Respiratory parameters				
FiO_2_ (%)	60 (50, 90)	41 (37, 41)	1259.000	**<0.001**
PEEP (cmH_2_O)	5 (5, 7)	0 (0, 0)	51.000	**<0.001**
Blood gas analysis				
pH	7.39 (7.30, 7.45)	7.42 (7.38, 7.46)	3010.000	**0.011**
PaO_2_ (mmHg)	66.0 (55.6, 77.7)	61.2 (54.1, 76.3)	3864.500	0.567
PaCO_2_ (mmHg)	37.2 (31.0, 47.0)	33.0 (27.5, 37.1)	2773.000	**0.002**
Lactic acid (mmol/L)	1.9 (1.3, 3.1)	1.9 (1.2, 2.5)	3670.000	0.308
Oxygenation Index (first time)	107.0 (70.5, 153.9)	165.5 (133.4, 186.0)	2113.000	**<0.001**
Oxygenation index (corrected)	138.0 (91.6, 200.1)	215.1 (173.4, 241.8)	2125.000	**<0.001**
Laboratory indicators				
SpO_2_/FiO_2_ (Pulse Oximeter)	145.0 (97.5, 192.5)	226.8 (206.0, 240.5)	1379.000	**<0.001**
SpO_2_/FiO_2_ (corrected)	188.5 (126.7, 250.2)	294.8 (267.9, 312.7)	1376.000	**<0.001**
Red blood cell count (×10^12^/L)	4.25 (3.31, 4.90)	3.57 (2.78, 4.60)	3083.500	**0.018**
White Blood Cell Count (×10^12^/L)	11.02 (7.38, 15.83)	8.73 (6.18, 11.11)	2864.500	**0.004**
Platelet count (×10^12^/L)	137.5 (88, 196.2)	132.5 (90.2, 214.1)	4006.500	0.805
Hemoglobin (g/L)	129.5 (102, 151.2)	114.0 (84.0, 138.2)	3145.000	**0.026**
Hematocrit (%)	38.3 (30.7, 46.0)	34.0 (27.5, 40.5)	3.158.500	**0.028**
Total Bilirubin (μmol/L)	20.4 (14.3, 32.4)	13.7 (10.9, 23.9)	2877.500	**0.005**
C-reactive protein (mg/L)	10.6 (3.8, 22.0)	8.2 (3.9, 15.0)	3453.500	0.130
Renal function indicators				
Creatinine level (μmol/L)	87 (66, 127)	84 (66, 156)	3935.500	0.682
Cystine (mg/L)	1.23 (0.98, 1.95)	1.24 (1.05, 2.20)	3784.500	0.450
Blood Urea Nitrogen (mmol/L)	8.60 (5.84, 13.63)	9.16 (5.27, 14.80)	4016.000	0.822
Disease scores				
APACHEII scores	23 (17, 29)	21 (14, 24)	3284.500	0.057
SOFA scores	8 (6, 11)	6 (4, 9)	3121.500	**0.022**
SIRS scores	2 (2, 3)	2 (1, 3)	3265.500	**0.041**
CURB-65 scores	2 (2, 3)	2 (2, 3)	3811.500	0.461
Hospitalization status				
ICU length of stay	7 (3, 16)	5.5 (3.0, 10.2)	3863.000	0.564
Risk factors for etiology				
Pneumonia (*n* = 152)	132 (86.8)	20 (13.2)	0.976^a^	0.807
Infection (*n* = 69)	61 (88.4)	8 (11.6)		
Aspiration (*n* = 6)	6 (100)	0 (0)		
Severe trauma (*n* = 49)	43 (87.8)	6 (12.2)		
Underlying medical conditions				
Diabetes (*n* = 64)	56 (87.5)	8 (12.5)	13.948^a^	0.787
Hypertension (*n* = 116)	107 (92.2)	9 (7.8)		
Chronic pulmonary insufficiency (*n* = 105)	94 (89.5)	11 (10.5)		
Chronic heart failure (*n* = 103)	88 (85.4)	15 (14.6)		
None (*n* = 57)	47 (82.5)	10 (17.5)		

The elevation of the Xining area is 2,261 m; Atmospheric pressure is approximately 581 ~ 582 mmHg; Perform calibration for PaO_2_/FiO_2_ or SpO_2_/FiO_2_ × 1.3; pH is the acidity or alkalinity level; PaO_2_ is the partial pressure of oxygen in arterial blood; PaCO_2_ is the partial pressure of carbon dioxide in arterial blood; SpO_2_ denotes blood oxygen saturation; FiO_2_ denotes inspired oxygen concentration; PEEP denotes positive end-expiratory pressure; the oxygenation index denotes PaO_2_/FiO_2_; Oxygenation index (corrected): PaO_2_/FiO_2_ (corrected); APACHE II scores (Acute Physiology and Chronic Health Evaluation II); SOFA scores (Sequential Organ Failure Assessment); SIRS scores (Systemic Inflammatory Response Syndrome, SIRS); CURB-65 Scores (CURB-65 Pneumonia Severity Scores);a for row × list χ^2^ test.

The bold values indicate *p* < 0.05.

### 3.3 Receiver operating characteristic curve evaluation using S/F instead of P/F to assess oxygenation in patients with ARDS with different subtypes

**Figure 2:**
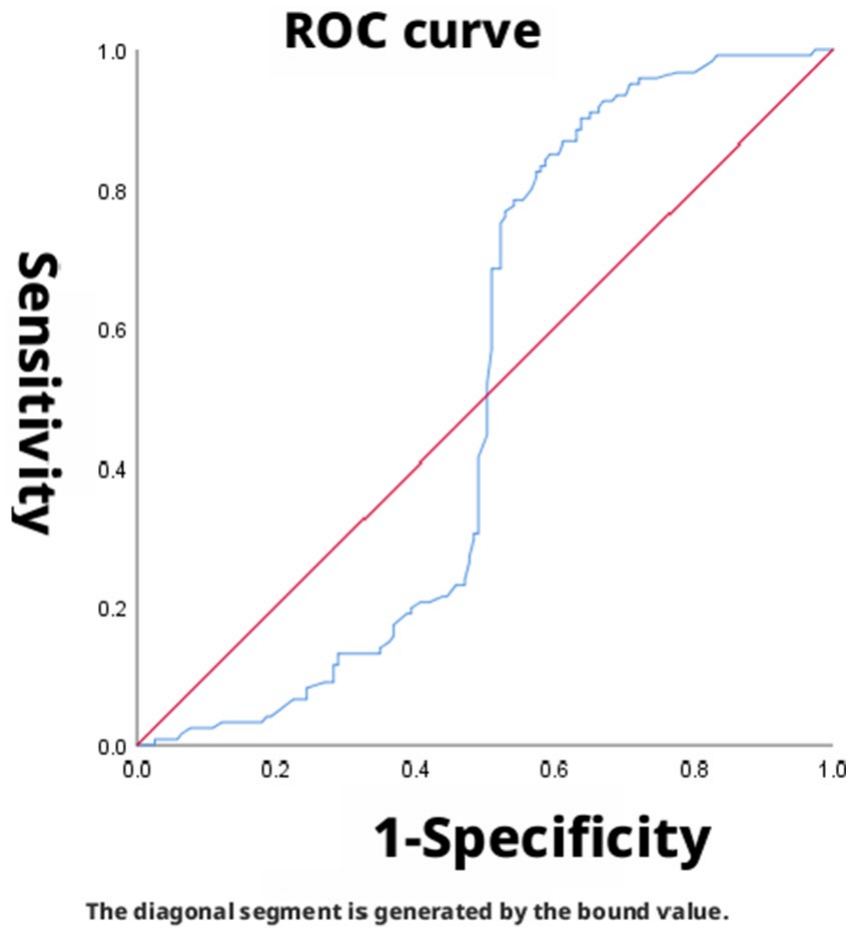
SpO_2_/FiO_2_ (corrected) predicted PaO_2_/FiO_2_ (corrected) ≤ 300 ROC curve, dashed lines represent 95% confidence intervals, AUC = 0.912.

**Figure 3:**
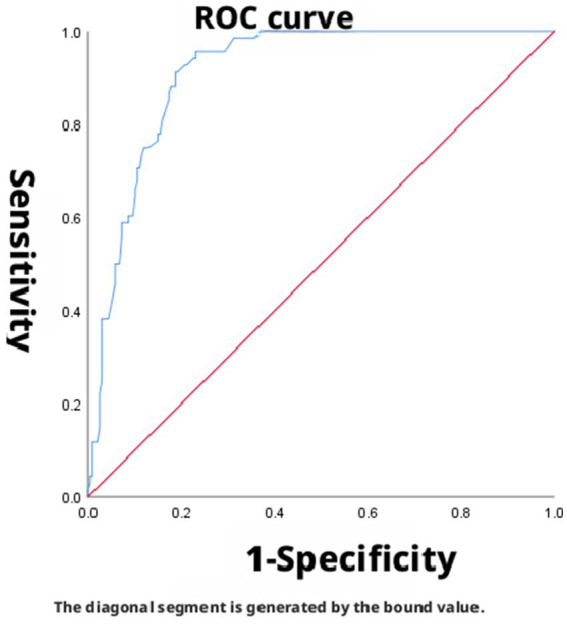
SpO_2_/FiO_2_ (corrected) predicted PaO_2_/FiO_2_ (corrected) ≤ 200 ROC curve, dashed lines represent 95% confidence intervals, AUC = 0.487.

**Figure 4:**
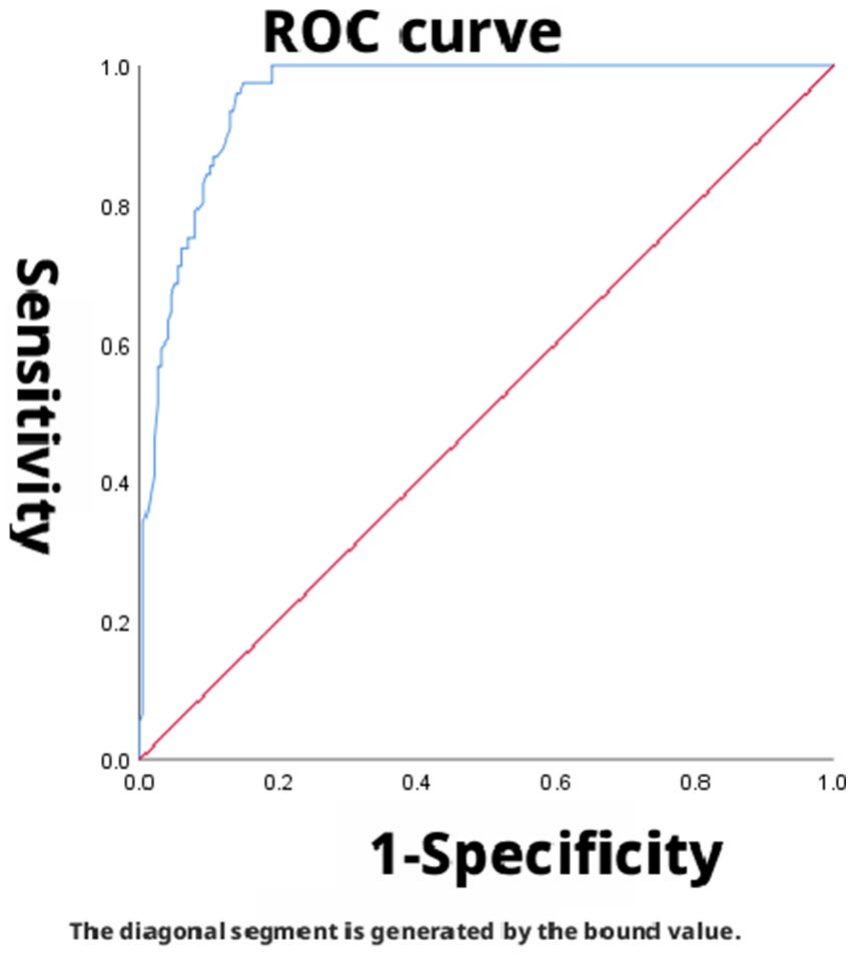
SpO_2_/FiO_2_ (corrected) predicted PaO_2_/FiO_2_ (corrected) ≤ 100 ROC curve, dashed lines represent 95% confidence intervals, AUC = 0.956.

The area under the curve (AUC) for SpO_2_/FiO_2_ (corrected) in predicting mild ARDS was 0.912 (95% CI: 0.879–0.945), indicating good discriminatory ability. The optimal cutoff value for predicting mild ARDS, determined by maximizing the Youden index, was 360.4. At this threshold, the sensitivity and specificity were 89.7 and 80.8%, respectively, with a corresponding Youden index of 0.705 (Figure 2). The AUC for SpO_2_/FiO_2_ (corrected) in predicting moderate ARDS was 0.487 (95% CI: 0.416–0.558), indicating low discriminatory ability. The Youden index was maximized at a cutoff of 238.8, yielding a sensitivity of 51.2% and a specificity of 94.8% (Youden index = 0.460) (Figure 3). The AUC for SpO_2_/FiO_2_ (corrected) in predicting severe ARDS was 0.956 (95% CI: 0.935–0.977), indicating good discriminatory ability. The optimal cutoff, identified by maximizing the Youden index, was 168, providing a sensitivity of 97.4% and a specificity of 85.0% (Youden index = 0.824) (Figure 4).

### 3.4 Correlation analysis between SpO_2_/FiO_2_ (corrected) and PaO_2_/FiO_2_ (corrected) and curve estimation

The correlation coefficient between SpO_2_/FiO_2_ (corrected) and PaO_2_/FiO_2_ (corrected) was *ρ* = 0.876 (strong positive correlation) (*p* < 0.001; *n* = 276). After controlling for confounding variables (Hemoglobin, PEEP, and Potential of hydrogen), partial correlation analysis revealed a strong correlation between SpO_2_/FiO_2_ (corrected) and PaO_2_/FiO_2_ (corrected) (*r* = 0.811, *p* < 0.001, *n* = 276). SpO_2_/FiO_2_ (corrected) and PaO_2_/FiO_2_ (corrected) showed a strong positive correlation.

**Figure 5:**
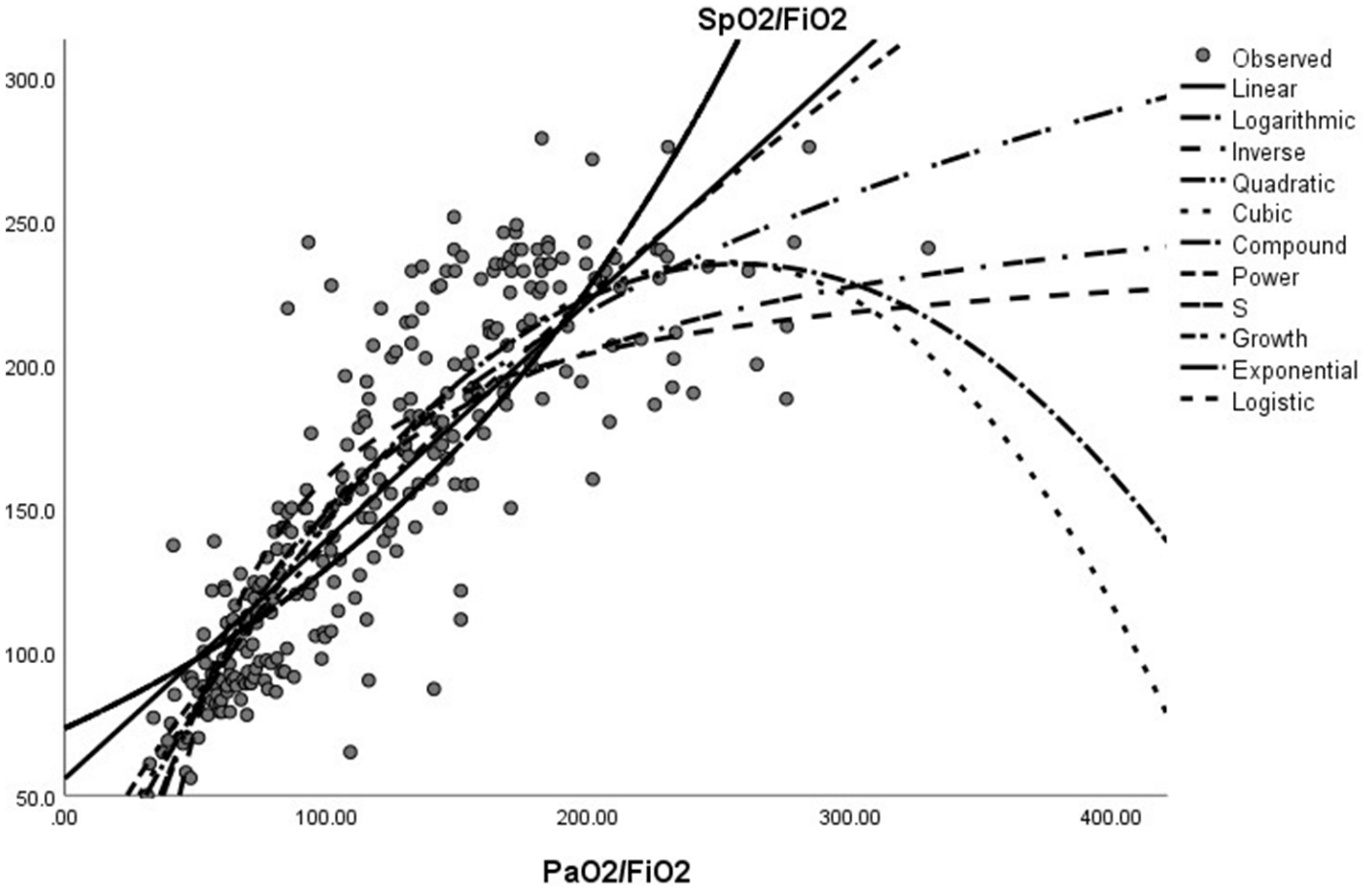
Estimated Curve of SpO_2_/FiO_2_ vs. PaO_2_/FiO_2_.

Among the models describing the relationship between SpO_2_/FiO_2_ (dependent variable) and PaO_2_/FiO_2_ (independent variable), the power model exhibited the highest R-squared (R^2^) value (0.759), followed by the cubic (0.757) and quadratic (0.756) models. The logarithmic (0.737), S (0.726), linear (0.688), composite/growth/exponential/logistic (0.666), and inverse (0.657) models exhibited lower R^2^ values. All models had an R^2^ value greater than 0.65, indicating that the basic fitting was effective. However, the power, cubic, and quadratic models were significantly superior, and all models were highly significant (*p* < 0.001), with the power model exhibiting an extremely high *F*-value (*F* = 860.737). Thus, the optimal nonlinear model was: SpO_2_/FiO_2_ = 5.377 × [PaO_2_/FiO_2_]^0.704^. The linear model of SpO_2_/FiO_2_ = 64 + 0.84 × (PaO_2_/FiO_2_) (*r* = 0.89, *p* < 0.0001) (11) was retained in the current analysis: SpO_2_/FiO_2_ = 55.835 + 0.831 × (PaO_2_/FiO_2_) (Appendix 1; Figure 5).

**Figure 6:**
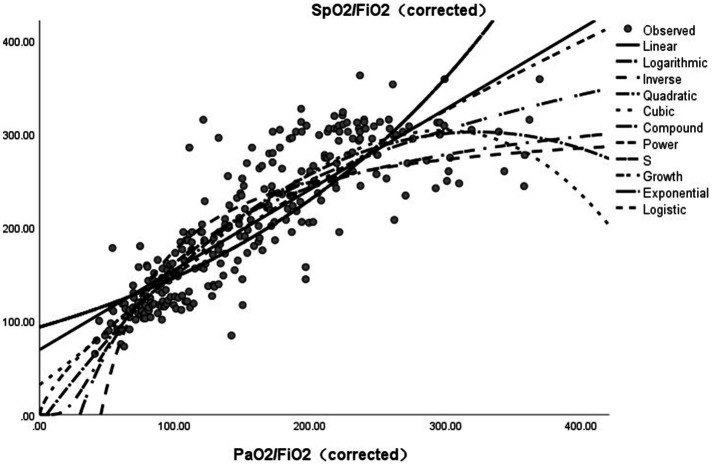
Estimated curve of SpO_2_/FiO_2_ (corrected) vs. PaO_2_/FiO_2_ (corrected).

In the relationship model between SpO_2_/FiO_2_ (corrected) (dependent variable) and PaO_2_/FiO_2_ (corrected), the highest R^2^ values were achieved using the cubic and power models (R^2^ = 0.769), followed by the quadratic (R^2^ = 0.766), logarithmic (R^2^ = 0.745), and S models (R^2^ = 0.732). Lower values were observed in the linear (R^2^ = 0.704) and inverse models (R^2^ = 0.662), as well as in the composite, growth, exponential, and logistic model groups (R^2^ = 0.685). All models achieved R^2^ values above 0.6, indicating acceptable fitting performance; however, significant differences existed among them, and the cubic and power models performed the best. R^2^ may overestimate the goodness-of-fit for complex models if the number of predictor variables (k) is not considered. Therefore, an adjusted R^2^ value was calculated. The power model had the highest adjusted R^2^ value (0.7682), indicating that it was optimal after accounting for model complexity. Although the cubic model shares the same R^2^ value, its adjusted R^2^ was slightly lower (0.7665) owing to the inclusion of more parameters (k = 3). The power model achieved the highest R^2^ (0.769) and adjusted R^2^ (0.7682) values, along with an exceptionally high F-statistic (913.303). The coefficient for the cubic term (b3 ≈ 0) was not significant, and the adjusted R^2^ was slightly lower; therefore, it is not recommended as a priority. The optimal nonlinear model was SpO_2_/FiO_2_ (corrected) = 5.661 × (PaO_2_/FiO_2_ [corrected])^0.71^. The following linear model was also retained: SpO_2_/FiO_2_ (corrected) = 69.333 + 0.857 × [PaO_2_/FiO_2_ [corrected]) (Appendix 2; Figure 6).

The linear and optimal nonlinear formulas for SpO_2_/FiO_2_ vs. PaO_2_/FiO_2_ and SpO_2_/FiO_2_ (corrected) vs. PaO_2_/FiO_2_ (corrected) were estimated in patients with ARDS. The primary recommendation from the global redefinition proposes using SpO_2_/FiO_2_ for ARDS severity grading when SpO_2_ ≤ 97%, with the following thresholds: mild (235 mmHg < SpO_2_/FiO_2_ ≤ 315 mmHg), moderate (148 mmHg < SpO_2_/FiO_2_ ≤ 235 mmHg), and severe (SpO_2_/FiO_2_ ≤ 148 mmHg). The corresponding SpO_2_/FiO_2_ critical values for guideline PaO_2_/FiO_2_ thresholds of 100, 200, and 300 were calculated using four separate formulas. Formula 1 yielded SpO_2_/FiO_2_ critical values of 137.6, 224.1, and 298.1, respectively; Formula 2 yielded SpO_2_/FiO_2_ critical values of 138.9, 222.0, and 305.1, respectively; Formula 3 yielded SpO_2_/FiO_2_ (corrected) thresholds of 148.9, 243.6, and 324.8, respectively; and Formula 4 yielded SpO_2_/FiO_2_ (corrected) thresholds of 155.0, 240.7, and 326.4, respectively (Table 3).

**Table 3:** SpO_2_/FiO_2_ values corresponding to different formulas at the guideline threshold.

Classification by severity	2023 Guide		SpO_2_/FiO_2_			
	PaO_2_/FiO_2_	SpO_2_/FiO_2_	剬式1	剬式2	剬式3	剬式4
Mild	300	315	298.1	305.1	324.8	326.4
Moderate	200	235	224.1	222.0	243.6	240.7
Severe	100	148	137.6	138.9	148.9	155.0

Formula 1 is a nonlinear model: SpO_2_/FiO_2_ = 5.377 × [PaO_2_/FiO_2_] ^0.704^; Formula 2 is a linear model: SpO_2_/ FiO_2_ = 55.835 + 0.831 × [PaO_2_/FiO_2_]; Formula 3 is a nonlinear mode: lSpO_2_/ FiO_2_ (校正后) = 5.661 × [PaO_2_/FiO_2_ (corrected)]^0.71^; Formula 4 is a linear modelSpO_2_/ FiO_2_ (corrected) = 69.333 + 0.857 × [PaO_2_/FiO_2_ (corrected)].

## 4 Discussion

The hypoxemia severity grouping thresholds based on the S/F ratios proposed by the new global definition of ARDS may not be applicable to the Xining region, and different grouping thresholds may exist owing to variations in altitude. The nonlinear relationship between SpO_2_/FiO_2_ and PaO_2_/FiO_2_ ratios identified in this study may better align with the characteristics of patients with ARDS in Xining, Qinghai Province.

ARDS is a heterogeneous syndrome with complex pathophysiology and mechanisms. Current ARDS treatment remains primarily supportive, focusing on managing the underlying disease, and novel pharmacological interventions are required (18). A study examining the effect of inhaled nitric oxide (iNO) on outcomes in patients with COVID-19-related ARDS indicated that exogenous iNO may improve oxygenation but does not reduce mortality rates in Acute lung injury/ARDS (19). In most ICUs, non-invasive and continuously available SpO_2_ monitoring is essential (11). Pulse oximeters offer continuous operation and are characterised by their accuracy, affordability, and non-invasive nature (1). The pulse oximetry index for ARDS has been widely adopted in pediatric medicine and in resource-limited settings. The new global definition of ARDS recommends using SpO_2_/FiO_2_ < 315, if ≤97%, to identify hypoxemia; therefore, the SpO_2_/FiO_2_ ratio can be considered a diagnostic tool for early admission to clinical trials (1, 20). For example, recent ARDS clinical trials have employed the SpO_2_/FiO_2_ ratio for patient selection, and patients diagnosed with ARDS based on SpO_2_/FiO_2_ exhibited similar clinical outcomes (21, 22). In recent years, non-invasive monitoring techniques have been increasingly applied in the field of respiratory critical care. Studies have demonstrated the promising potential of exhaled breath volatile organic compound (VOC) analysis as a non-invasive screening tool for early lung cancer detection (23). Concurrently, oxygen assessment methods based on pulse oximetry have proven to be of significant value in clinical practice. The continued advancement of these technologies offers new possibilities for the early identification and dynamic monitoring of acute respiratory distress syndrome (ARDS), particularly in resource-limited settings.

High-altitude regions are characterized by low oxygen pressures, intense radiation, and cold climates (6, 9). As altitude increases, atmospheric pressure and the partial pressure of inhaled oxygen decrease (24). High-altitude ARDS exhibits more pronounced changes in pathophysiological alterations, clinical symptoms, signs, and blood gas parameters than low-altitude ARDS. This changes is likely caused by the combined effects of increasing altitude gradients, decreasing partial pressure of oxygen gradients, and exposure to high-altitude environmental factors, all of which affect the underlying etiology, pathology, and physiology of ARDS (3). Individuals who have lived at high altitudes for extended periods exhibit varying degrees of tolerance to hypoxic environments, resulting in distinct pathophysiological changes in patients with ARDS at high altitudes compared to those in lowland areas (9). Compared with low-altitude ARDS, high-altitude ARDS is characterized by a combination of low pressure and hypoxia, faster disease progression, poorer prognosis, need for correction of the oxygenation index (760/local atmospheric pressure × measured value), more severe pulmonary exudation, more pronounced hypoxemia, increased susceptibility to acute cor pulmonale, higher pulmonary artery pressure, lower PEEP settings, longer prone position ventilation duration, and more challenging and relatively conservative volume management (3). This study excluded patients who met the diagnostic criteria for high-altitude polycythemia (HAPC; hemoglobin >210 g/L in males and >190 g/L in females). While this exclusion was intended to mitigate the confounding effects of this pathological condition on the physiological relationship between SpO_2_ and PaO_2_, it consequently means that our findings are primarily applicable to native high-altitude residents with normal hemoglobin levels. Therefore, caution should be exercised when extrapolating these conclusions to ARDS patients with concomitant HAPC. In healthy participants, changes in PaO_2_ and SpO_2_ show a strong correlation when pulse oximetry readings fall within the 80–100% range.

In this study, the AUCs for predicting mild and severe ARDS using SpO_2_/FiO_2_ (corrected) were 0.912 and 0.956, respectively. SpO_2_/FiO_2_ (corrected) demonstrated good discriminatory ability for both mild and severe ARDS. However, the AUC for SpO_2_/FiO_2_ (corrected) in predicting moderate ARDS was 0.487, indicating low discriminatory ability. These results indicate that SpO_2_/FiO_2_ (corrected) effectively distinguishes patients with mild and severe ARDS but has limited diagnostic value for moderate ARDS. Its optimal diagnostic cutoff points were 360.4 for mild, 238.8 for moderate, and 168 for severe ARDS, which differ from those of the newly defined SpO_2_/FiO_2_ severity grading system (mild: 315, moderate: 235, and severe: 148). This discrepancy may stem from the lack of conclusive evidence regarding the suitability of the “newly defined SpO_2_/FiO_2_ correction standard” for patients with ARDS in high-altitude regions (9). From the perspective of high-altitude pathophysiological characteristics, the poor diagnostic performance of the SpO_2_/FiO_2_ (corrected) for moderate ARDS has a deeper physiological basis. At the pathophysiological level, patients with moderate ARDS typically have PaO_2_ values in the range of 60–80 mmHg, which falls precisely on the steep portion of the oxygen-hemoglobin dissociation curve. In this range, SpO_2_ is relatively insensitive to changes in PaO_2_. Even a substantial decrease in PaO_2_ (e.g., from 80 mmHg to 60 mmHg) may result in only a modest decline in SpO_2_ (e.g., from 96 to 90%), as the blood can still carry sufficient oxygen without manifesting overt hypoxemia. This physiological buffering effect may lead to very low resolution of SpO_2_-based indices in the moderate oxygenation range, thereby yielding an AUC approaching that of random guessing (0.5). Furthermore, long-term high-altitude residents exhibit elevated levels of 2,3-diphosphoglycerate (2,3-DPG), which shifts the oxygen-hemoglobin dissociation curve to the right. This effect is most pronounced in the transitional zone of the curve-precisely the region corresponding to moderate ARDS-resulting in increased inter-individual variability in SpO_2_ at the same PaO_2_ level. Compounding this, native high-altitude residents display considerable inter-individual differences in their adaptation to hypoxia (e.g., ventilatory compensation, erythrocyte proliferation). While these differences may be masked in the relatively stable oxygenation ranges of mild and severe ARDS, they become markedly amplified in the critical zone of moderate ARDS. Another consideration relates to overlap between severity groups. There is an inherent physiological continuum between mild and moderate, and between moderate and severe ARDS. Although the Berlin definition imposes artificial boundaries, patients with values near the thresholds (e.g., PaO_2_/FiO_2_ close to 200 or 100) often exhibit overlapping clinical presentations in practice. Our data also show that, while the AUCs for the mild and severe groups were excellent, the confidence interval for the moderate group (95% CI: 0.416–0.558) completely crossed the line of no discrimination, indicating a potentially high rate of misclassification. In this subset of patients, accurate assessment must rely on arterial blood gas analysis. Thus, the SpO_2_/FiO_2_ (corrected) cannot serve as a complete substitute for PaO_2_/FiO_2_ in all ARDS patients. Its clinical value lies primarily in the rapid screening and identification of patients at the extremes of severity-namely, those with mild or severe ARDS.

Owing to the shape of the oxyhemoglobin dissociation curve, the correlation between SpO_2_ and PaO is highest when SpO_2_ values were 97% or lower (5). Patients with SpO_2_ values exceeding 97% and those at altitudes above 1,000 meters were excluded when initially deriving and validating the PaO_2_/FiO_2_ ratio from the SpO_2_/FiO_2_ ratio (11). Low atmospheric pressure in high-altitude regions also affects the relationship between PaO_2_ and SpO_2_ (5). SpO_2_/FiO_2_ and PaO_2_/FiO_2_ demonstrated strong linear relationships in patients with moderate or severe ARDS; therefore, risk stratification using SpO_2_/FiO_2_ and PEEP may be practical, particularly in resource-limited settings (25). A quadratic function model in a 2017 study described the relationship between SpO_2_/FiO_2_ and PaO_2_/FiO_2_ in patients receiving mechanical ventilation or with ARDS compared to a linear model. In a 2016 study, the performance of linear, log-linear, and nonlinear equations used in the current study was compared, demonstrating that the nonlinear relationship of PaO_2_/FiO_2_ based on the Severinghaus equation outperformed the linear and log-linear equations in patients with ARDS. Given the S-shaped relationship between SpO_2_ and PaO_2_, employing a nonlinear equation strategy appears to be more advantageous than a linear equation strategy (12). The Severinghaus–Ellis (nonlinear), Rice (linear), and Pandharipande (log-linear) formulas demonstrated high consistency in estimating noninvasive oxygenation indices from SpO_2_ calculations compared to PaO_2_/FiO_2_ values obtained via arterial blood gas analysis. Additionally, the nonlinear formula yielded superior diagnostic accuracy for moderate hypoxemia (14). The nonlinear model: SpO_2_/FiO_2_ (corrected) = 5.661 × [PaO_2_/FiO_2_ (corrected)]^0.71^ aligns the best with the Xining region of Qinghia Province, as its values are the closest to the stratified values in the 2023 ARDS guidelines. The linear model obtained in this study is similar to the formula SpO_2_/FiO_2_ = 45.614 + 0.847 × [PaO_2_/FiO_2_ (corrected)] used by Wang et al. (26). However, the resulting SpO_2_/FiO_2_ stratification values for ARDS exhibit certain discrepancies. Furthermore, the nonlinear equation strategy is preferable to the linear equation strategy (12). Ultimately, the optimal nonlinear model estimated from the curve was retained, namely, Formula 3. This nonlinear formula may be used for severity grading in patients from the Xining area of Qinghai Province who lack access to invasive blood gas analyses. Alternatively, hypoxemia can be identified by measuring the patient’s oxygen saturation using a noninvasive pulse oximeter, followed by invasive blood gas analysis to determine the severity of the condition.

However, this study had certain limitations. First, it primarily enrolled patients with ARDS from the Department of Critical Care Medicine at Qinghai Provincial People’s Hospital. Although this institution admits patients from across the province, it cannot completely eliminate the inherent selection bias and geographic limitations associated with a single-center study. The model developed in this study is exploratory in nature, and its generalizability requires validation using independent external datasets. Future research should involve multi-center studies with larger sample sizes to assess the model’s transportability and to explore whether model calibration is necessary based on different population characteristics. Second, the blood gas analysis and clinical data collected represent only the initial results after ICU admission, lacking continuous monitoring across multiple time points. Assessing hypoxemia severity at a single time point may lead to the misclassification of patients, as their condition can dynamically improve and deteriorate within a single day (12). To address this limitation, we plan to design a prospective cohort study involving continuous monitoring of ARDS patients at standardized time points. Arterial blood gas and clinical data will be collected at predefined intervals, including 0 h, 24 h, and 48 h after ICU admission, for further investigation. Third, the primary focus of this study was to validate the diagnostic consistency between the SpO_2_/FiO_2_ (corrected) and the PaO_2_/FiO_2_ (corrected). However, we have not yet performed an independent prognostic evaluation of the derived optimal cutoff values for predicting clinical outcomes, nor have we thoroughly investigated the causal relationships between adjustable therapeutic factors such as PEEP and patient outcomes. These limitations are largely attributable to the single-center retrospective design, including its sample size constraints and the inherent nature of the data. To address these gaps, we plan to conduct a multi-center, large-sample prospective cohort study. This future research will take patient-centered outcomes (e.g., 28-day mortality and duration of mechanical ventilation) as the primary endpoints to validate the prognostic value of the SpO_2_/FiO_2_ stratification thresholds proposed for high-altitude regions. Furthermore, drawing on the target trial emulation (TTE) framework articulated by Yang et al., we aim to move observational data toward causal inference by defining a clear “target trial”—for instance, comparing high versus low PEEP strategies in terms of their effect on 28-day mortality in ARDS patients (27). Such efforts will provide a more robust evidence base for precision treatment of ARDS in high-altitude settings. Four, another important limitation of this study is the insufficient consideration of the inherent heterogeneity of the ARDS population. ARDS is a highly heterogeneous clinical syndrome, characterized by significant inter-individual variability in etiology, pathophysiological processes, and treatment responses. By pooling patients with different etiologies, underlying diseases, and ventilation modes, our analysis may have masked the true diagnostic performance of certain subgroups or overestimated the applicability of the index in others. As articulated by Yang et al. in their work on clinical subphenotype identification, future research should employ data-driven clustering approaches to explore potential subtypes of ARDS (28). Conducting stratified analyses by etiology—for instance, separately examining patients with ARDS secondary to pneumonia, extrapulmonary infection, and trauma—would help determine whether the diagnostic performance of the SpO_2_/FiO_2_ ratio is etiology-specific. Such an approach holds promise for more precisely defining the target population and application boundaries of the SpO_2_/FiO_2_ ratio in high-altitude ARDS patients, thereby providing a foundation for precision diagnosis and individualized treatment of ARDS.

## 5 Conclusion

In this study, clinical data from 276 patients revealed a nonlinear relationship between pulse oximetry SpO_2_/FiO_2_ and PaO_2_/FiO_2_ ratios (power function model: SpO_2_/FiO_2_ (corrected) = 5.661 × [PaO_2_/FiO_2_ (corrected)]^0.71^), providing a model basis for substituting SpO_2_/FiO_2_ for PaO_2_/FiO_2_. SpO_2_/FiO_2_ exhibited excellent predictive efficacy for mild and severe ARDS, with cutoff values of 360.4 for mild and 168 for severe ARDS, respectively, enabling precise diagnostic stratification. However, its predictive efficacy for moderate ARDS remained limited. This study innovatively highlights that globally redefined SpO_2_/FiO_2_-based thresholds may not be applicable in high-altitude regions, such as Xining, suggesting that altitude variations should be considered when revising the diagnostic criteria. Through model construction and validation, this study expands hypoxemia assessment methods, providing scientific evidence for early identification of ARDS and precise treatment in resource-constrained areas and high-altitude environments, while emphasizing the importance of regionalized diagnostic thresholds. Future research may need to incorporate altitude differences into the revision of diagnostic criteria, develop corresponding models and validate them, thereby providing scientific basis for the early identification and precise treatment of ARDS in high-altitude environments.

## Data Availability

The raw data supporting the conclusions of this article will be made available by the authors, without undue reservation.

## Footnotes

1
https://www.xining.gov.cn/zjxn/xngk/ly/wzjy/202102/t20210201_72938.html
