# Supplementary material for: Relationship between SpO2/FiO2 and PaO2/FiO2 in patients with acute respiratory distress syndrome: a single-center, retrospective cohort study from Xining, China
Source: Front Med (Lausanne). 2026 May 7;13:1774390. doi: 10.3389/fmed.2026.1774390 (PMC13190466; doi:10.3389/fmed.2026.1774390)
Supplement: Supplementary file 1 [file Table_1.DOCX]

Appendix Table1：Summary and Parameter Estimation of SpO₂/FiO₂ and PaO₂/FiO₂ Models

| Equation | Model Summary | | | | | Parameter estimates | | | |
| --- | --- | --- | --- | --- | --- | --- | --- | --- | --- |
|  | R ^2^ | F | Degree of Freedom 1 | Degree of Freedom 2 | Significance | Constant | b1 | b2 | b3 |
| Linear | 0.688 | 603.694 | 1 | 274 | ＜0.001 | 55.835 | 0.831 |  |  |
| Logarithm | 0.737 | 769.705 | 1 | 274 | ＜0.001 | -314.498 | 100.572 |  |  |
| inverse | 0.657 | 525.98 | 1 | 274 | ＜0.001 | 246.799 | -8626.879 |  |  |
| quadratic | 0.756 | 423.005 | 2 | 273 | ＜0.001 | -3.889 | 1.857 | -0.004 |  |
| cubic | 0.757 | 281.765 | 3 | 272 | ＜0.001 | 6.387 | 1.591 | -0.002 | -3.98E^-6^ |
| composite | 0.666 | 545.832 | 1 | 274 | ＜0.001 | 73.377 | 1.006 |  |  |
| power | 0.759 | 860.737 | 1 | 274 | ＜0.001 | 5.377 | 0.704 |  |  |
| S | 0.726 | 725.883 | 1 | 274 | ＜0.001 | 5.633 | -62.564 |  |  |
| growth | 0.666 | 545.832 | 1 | 274 | ＜0.001 | 4.296 | 0.006 |  |  |
| exponential | 0.666 | 545.832 | 1 | 274 | ＜0.001 | 73.377 | 0.006 |  |  |
| Logistic | 0.666 | 545.832 | 1 | 274 | ＜0.001 | 0.014 | 0.994 |  |  |
