# Supplementary material for: Relationship between SpO2/FiO2 and PaO2/FiO2 in patients with acute respiratory distress syndrome: a single-center, retrospective cohort study from Xining, China
Source: Front Med (Lausanne). 2026 May 7;13:1774390. doi: 10.3389/fmed.2026.1774390 (PMC13190466; doi:10.3389/fmed.2026.1774390)
Supplement: Supplementary file 2 [file Table_2.DOCX]

Appendix Table2：Model Summary and Parameter Estimates for SpO₂/FiO₂ (Corrected) and PaO₂/FiO₂ (Corrected)

| Equation | Model Summary | | | | | Parameter estimates | | | |
| --- | --- | --- | --- | --- | --- | --- | --- | --- | --- |
|  | R ^2^ | F | Degree of Freedom 1 | Degree of Freedom 2 | Significance | Constant | b1 | b2 | b3 |
| Linear | 0.704 | 651.438 | 1 | 274 | ＜0.001 | 69.333 | 0.857 |  |  |
| Logarithm | 0.745 | 802.38 | 1 | 274 | ＜0.001 | -446.818 | 131.624 |  |  |
| inverse | 0.662 | 535.568 | 1 | 274 | ＜0.001 | 320.767 | -14492.637 |  |  |
| quadratic | 0.766 | 445.728 | 2 | 273 | ＜0.001 | -9.572 | 1.927 | -0.003 |  |
| cubic | 0.769 | 302.579 | 3 | 272 | ＜0.001 | 31.956 | 1.066 | 0.002 | -8.58E^-6^ |
| composite | 0.685 | 595.343 | 1 | 274 | ＜0.001 | 93.604 | 1.004 |  |  |
| power | 0.769 | 913.303 | 1 | 274 | ＜0.001 | 5.661 | 0.71 |  |  |
| S | 0.732 | 747.111 | 1 | 274 | ＜0.001 | 5.896 | -80.933 |  |  |
| growth | 0.685 | 595.343 | 1 | 274 | ＜0.001 | 4.539 | 0.004 |  |  |
| exponential | 0.685 | 595.343 | 1 | 274 | ＜0.001 | 93.604 | 0.004 |  |  |
| Logistic | 0.685 | 595.343 | 1 | 274 | ＜0.001 | 0.011 | 0.996 |  |  |
